# Biodiesel Processing Using Sodium and Potassium Geopolymer Powders as Heterogeneous Catalysts

**DOI:** 10.3390/molecules25122839

**Published:** 2020-06-19

**Authors:** Renata F. Botti, Murilo D.M. Innocentini, Thais A. Faleiros, Murilo F. Mello, Danilo L. Flumignan, Leticia K. Santos, Giorgia Franchin, Paolo Colombo

**Affiliations:** 1 Department of Industrial Engineering, University of Padova, via Marzolo 9, 35131 Padova, Italy; giorgia.franchin@unipd.it (G.F.); paolo.colombo@unipd.it (P.C.); 2 Course of Chemical Engineering, University of Ribeirão Preto (UNAERP), 14096-900 Ribeirão Preto, São Paulo, Brazil; minnocentini@unaerp.br (M.D.M.I.); thaisfaleiros1999@hotmail.com (T.A.F.); murilofmello@hotmail.com (M.F.M.); 3 Institute of Chemistry, Center for Monitoring and Research of the Quality of Fuels, Biofuels, Crude Oil and Derivatives–CEMPEQC, São Paulo State University (UNESP), 14800-900 Araraquara, São Paulo, Brazil; flumignan@ifsp.edu.br (D.L.F.); leticiaksantos@yahoo.com (L.K.S.); 4 Mato Grosso Federal Institute of Education, Science and Technology-Campus Cuiabá, Rua Profa. Zulmira Canavarros, 95, Centro, 78005-200 Cuiabá, Mato Grosso, Brazil; 5 Department of Materials Science and Engineering, The Pennsylvania State University, University Park, State College, PA 16802, USA

**Keywords:** heterogeneous catalyst, biodiesel, geopolymer, alkali, transesterification

## Abstract

This work investigates the catalytic activity of geopolymers produced using two different alkali components (sodium or potassium) and four treatment temperatures (110 to 700 °C) for the methyl transesterification of soybean oil. The geopolymers were prepared with metakaolin as an aluminosilicate source and alkaline activating solutions containing either sodium or potassium in the same molar oxide proportions. The potassium-based formulation displayed a higher specific surface area and lower average pore size (28.64–62.54 m²/g; 9 nm) than the sodium formulation (6.34–32.62 m²/g; 17 nm). The reduction in specific surface area (SSA) after the heat treatment was more severe for the sodium formulation due to the higher thermal shrinkage. The catalytic activity of the geopolymer powders was compared under the same reactional conditions (70–75 °C, 150% methanol excess, 4 h reaction) and same weight amounts (3% to oil). The differences in performance were attributed to the influences of sodium and potassium on the geopolymerization process and to the accessibility of the reactants to the catalytic sites. The Na-based geopolymers performed better, with FAME contents in the biodiesel phase of 85.1% and 89.9% for samples treated at 500 and 300 °C, respectively. These results are competitive in comparison with most heterogeneous base catalysts reported in the literature, considering the very mild conditions of temperature, excess methanol and catalyst amount and the short time spent in reactions.

## 1. Introduction

The continuous depletion of global petroleum reserves and the growing health and environmental concerns about burning fossil fuels have stimulated the research of alternative energy resources. In the case of diesel engines, the progressive adoption of more stringent exhaust emission standards and the pressure of the market have forced countries to find renewable fuel substitutes with similar or improved performance but with lower prices and a lower impact on the environment. Biodiesel is one of the renewable and biodegradable substitutes for diesel, produced from vegetable and algae oils or animal fats and with a chemical composition that results in a similar power performance when burned in diesel engines [1,2,3]. Biodiesel offers other advantages: it has little or no sulfur content, it mixes in all proportions with petro-diesel and it yields lower hydrocarbons, CO and particle exhaust emissions. The large variety of sources from which biodiesel can be produced has encouraged countries to find their own large-scale feedstock; for instance, rapeseed in Europe, soybean and lard in Brazil and the USA and palm and jathropa in Asia [4].

Biodiesel processing technology at an industrial scale is well established nowadays. The main route is the homogenous catalytic transesterification of the triglycerides present in oils and fats, which are converted together with short-chain alcohols (methanol or ethanol) into fatty acid alkyl esters (biodiesel) and a by-product (glycerol). The reaction is typically catalyzed by a strong alkali alcoholic solution, generally sodium or potassium alkoxides (prepared from Na or K sources), and this becomes soluble in the final reactional mixture. An excess of alcohol is required in order to shift the set of the three reversible reactions required to produce the maximum amount of biodiesel. Consequently, one of the most important processing steps is biodiesel purification, which removes not only the excess alcohol, the formed glycerol and the traces of unreacted glycerides and soaps but also all the alkali metal catalysts (Na or K) which become solubilized in the biodiesel [5]. Several international standards define the maximum contents of these contaminants in biodiesel (e.g., ASTM D6751 in the US, EN 14214 in Europe and RANP n°45/2014 in Brazil). There are two common industrial methods for purifying biodiesel: wet and dry washing. Both methods have many disadvantages, including increased processing time and cost because of the use of additional chemicals (water, ion-exchange resins, adsorbents, etc.) and the wastewater treatment.

The search for heterogeneous catalysts that could be more easily separated from the reaction mixture without compromising the time and costs for biodiesel processing has been intensified in recent years. Alkaline or alkaline earth oxides, alkali metals supported on alumina or zeolite, calcined hydrotalcite and ion-exchange resins are some examples of heterogeneous catalyst candidates that have been investigated [6,7,8,9,10].

Geopolymers are synthetic materials with a high porosity and specific surface area, and they have a chemical composition comparable to that of a zeolite; they are obtained by the reaction of an aluminosilicate source with a highly concentrated alkali hydroxide and/or silicate solution, leading to the formation of a three-dimensional continuous network that consolidates at a low temperature. They can be shaped into different forms, including powders, pellets, foams and 3D printed scaffolds; it is possible to customize them depending on the application. The microstructure of geopolymers is temperature dependent: an amorphous structure is present at low temperatures, while heat treatments above ~500–700 °C lead to (partially) crystalline structures [11]. Geopolymers have been recently investigated as potential heterogeneous catalysts for biodiesel production [12,13,14,15]. The catalyst’s specific surface area is relevant for the reactions using heterogeneous catalysts because it represents the catalytic site’s accessibility, and for mesoporous materials, the surface area can be large. The precise catalytic mechanism and the kinetic of transesterification provided by the geopolymers were not investigated in the present work. However, we can posit that their behavior is similar to that which is already well described in the literature for strongly alkaline porous solids [16,17]. For these heterogeneous catalysts, the catalytic mechanism involves a sequence of steps in which the absorption of methanol on the solid at alkaline sites is the rate determining step to produce the methoxide ion, which is in fact the actual catalyst that is active in the transesterification reaction.

Although the catalytic activity of geopolymers has already been reported in the literature, there are still some unclear issues concerning the influence of the formulation and processing conditions of geopolymers on the conversion of vegetable oils into biodiesel. In this work, geopolymer powders with formulations containing either sodium or potassium, which were heat treated in a broad range of temperatures, were produced, and their heterogeneous catalytic activity was evaluated for the transesterification of soybean oil and methanol in order to produce fatty acid methyl esters (biodiesel).

## 2. Results and Discussion

### 2.1. Characterization of Sodium and Potassium Geopolymers

**Figure 1:**
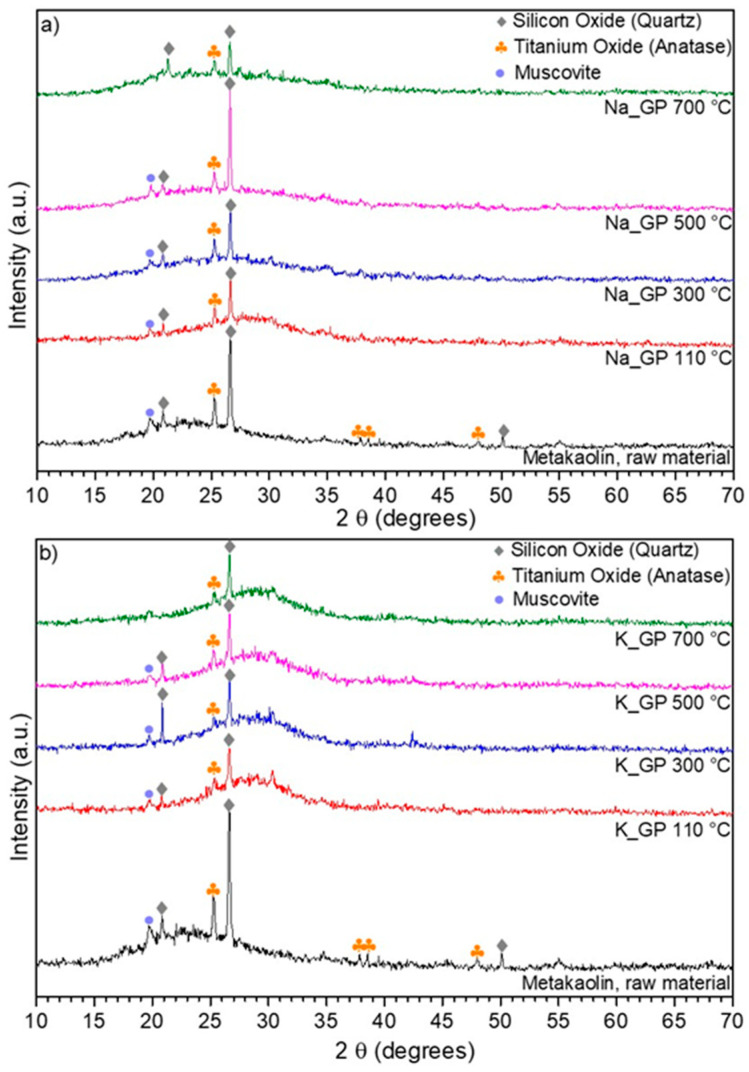
XRD patterns for metakaolin and geopolymer samples heat-treated at different temperatures: (**a**) Na-GP; (**b**) K-GP.

Figure 1 shows the XRD patterns for metakaolin (Argical M 1200S) and the geopolymer powders (sodium-based geopolymer, Na-GP and potassium-based geopolymer, K-GP) prepared for the study. The metakaolin pattern presented the typical amorphous hump for 2θ = 15–30°, while the most evident peaks, between 20° and 25°, corresponded to the crystalline forms of silicon oxide (quartz), titanium oxide (anatase) and muscovite. In Figure 1a,b, we can observe that samples Na-GP and K-GP, treated at four temperatures (110, 300, 500 and 700 °C), possessed completely amorphous structures: the only peaks detected were derived from impurities contained in the metakaolin raw material and corresponded to the above mentioned crystalline phases of quartz (2θ = 20.70° and 26.50°), anatase (2θ = 25.14°) and muscovite (2θ = 19.60°). Such impurities do not take part in the geopolymerization reaction, so they remain in the final geopolymer’s composition [18].

**Figure 2:**
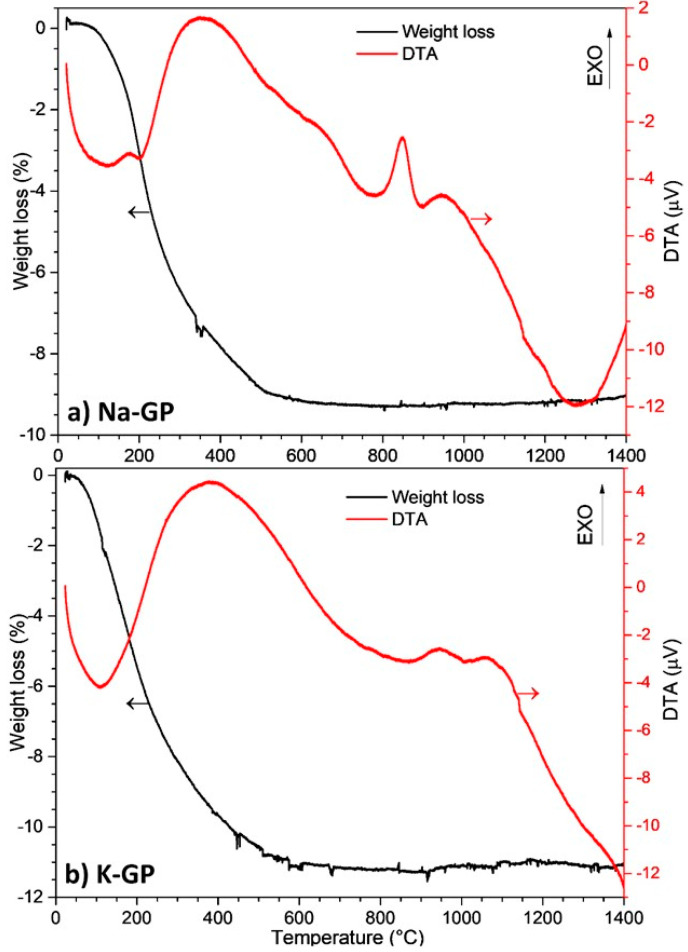
Thermal behavior of geopolymer samples: (**a**) Na-GP; (**b**) K-GP.

Figure 2 shows the results of the thermal analysis performed on the geopolymers after curing. For the Na-GP, there is an endothermic peak at approximately 110 °C, corresponding to the removal of physical water (Figure 2a). An exothermic peak is clear at around 350 °C, corresponding to the polycondensation into siloxo bonds of the unreacted phase in the geopolymer powder [11]. At around 850 °C, we can observe another exothermic peak, corresponding to the crystallization of the geopolymer phase. Crystals of nepheline (NaAlSiO_4_) usually form at this temperature. As the Na-GP powders were only treated up to 700 °C, it was confirmed that they did not develop any relevant crystalline phase. At 1300 °C, a small exothermic peak is present, probably due to the partial melting of the material. It is possible to correlate the first peaks of the DTA (Differential thermal analysis) curve with the largest weight loss, corresponding to approximately 9% at 550 °C, attributed to the release of water species within the tridimensional geopolymeric network. The total weight loss was 9.3% and remained constant with increasing temperature.

Figure 2b presents the DTA and weight loss profiles for sample K-GP. There is an endothermic peak at ~110 °C and an exothermic peak at ~380 °C, correlated to the loss of physically absorbed water and to the polycondensation into siloxo bonds, respectively. The weight loss reached 10.8% at 540 °C. At 950 °C, the DTA curve had an exothermic peak, corresponding to the formation of crystals of leucite (KAlSi_2_O_6_) and kalsilite (KAlSiO_4_) [19]. Additionally, in this case, the analysis corroborates the XRD data (Figure 1b), showing no development of crystalline phases in the K-GP powders up to 700 °C. The total weight loss for the K-GP was 11.3%, in agreement with values reported in the literature [20] and above the total water loss presented by the Na-GP (9.3%). The reason is that K has a greater molar mass (39 g/mol) than Na (23 g/mol), so greater amounts of potassium and chemically bonded water were introduced into the K-GP formulation, despite the same target molar proportions of K and Na.

Table 1 reports the BET (Brunauer–Emmett–Teller) and BJH (Barrett–Joyner–Halenda) parameters obtained from the N_2_ absorption tests for both geopolymers as a function of the heat treatment temperature. The specific surface area (SSA) of the Na-GP and K-GP samples decreased from 32.62 to 6.34 m²/g and from 62.54 to 28.64 m²/g, respectively, with an increase in heating temperature from 110 °C to 700 °C. Similar ranges are reported in the literature for sodium- and potassium-based geopolymers produced from metakaolin [21,22,23].

The consistently higher SSA and lower pore size values for the K-based geopolymer, as observed in Table 1, are in agreement with the trends shown in the literature [24,25]. The explanation for this has been discussed in detail by other authors [26,27]: it is related to the fact that potassium-activating solutions are less viscous and more alkaline than sodium solutions, facilitating the mass transfer, dissolution and reaction of metakaolin before hardening, with the consequent generation of a structure with smaller pores and a higher surface area. On the other hand, the decrease in the SSA and the total pore volume with an increasing treatment temperature is related to the reduction in the fraction of mesopores during the densification of the amorphous matrix, caused by thermal shrinkage.

Compared to other heterogeneous catalysts for biodiesel processing which have been described in the literature, both Na-GP and K-GP presented similar or higher SSA values. Maneerung et al. [28] obtained CaO from chicken manure. The catalyst was calcined at 550 and 950 °C and the specific surface areas were 11.2 and 10.0 m^2^/g, respectively. Pandit et al. [29] synthesized a CaO catalyst from natural waste material and calcined it at 900 °C. The study reported an SSA of 15.73 m^2^/g. Du et al. [30] utilized carbon-based MgO catalysts for biodiesel production from castor oil and reported SSAs of 8.65 and 13.26 m^2^/g for the catalyst calcined at 600 and 800 °C, respectively.

**Figure 3:**
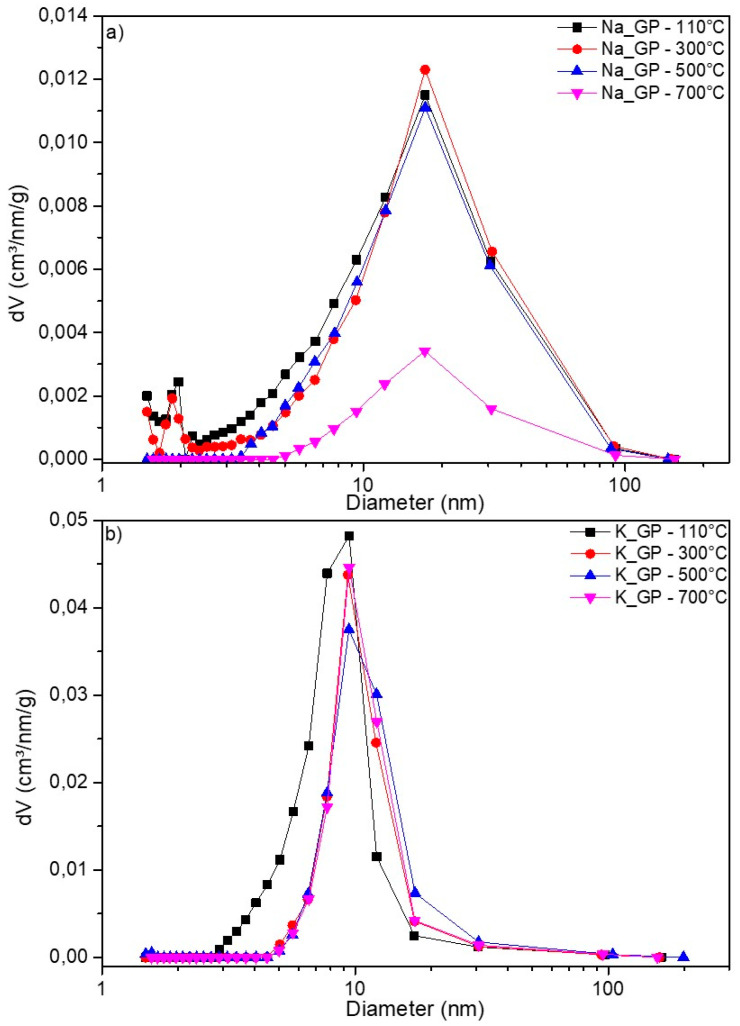
Pore distribution for samples: (**a**) Na-GP; (**b**) K-GP.

It is worth noting that the mesoporosity of geopolymer materials is due to the polymerization reaction (polycondensation of aluminosilicates), which produces water that is later removed by evaporation. At the nanometer scale, the porosity is due to the morphology of the phases, which is based on the aggregation of spheroidal nano-sized particles [11]. The pore size distributions of the Na-GP and K-GP geopolymers are shown in Figure 3 and corroborate the data in Table 1. Sample Na-GP presented mesopores (2 nm < diameter < 50 nm), with an average pore diameter of ~17 nm and a small amount of micropores (diameter < 2 nm). However, after the heat treatment at 700 °C, the sample lost most of its micro- and meso-porosity. The total pore volume decreased from 0.3 to 0.08 cm^3^/g, (Table 1), in agreement with the decrease in SSA, as previously discussed. The sample K-GP maintained a total pore volume of ~0.3 cm^3^/g with the increasing temperature, since potassium promotes lower thermal shrinkage than sodium; nevertheless, the K-GP sample had a smaller average pore diameter, at ~9 nm, with no micropores present (Figure 3b).

It is important to emphasize the fact that SSA and pore size are key features that affect the activity of a catalyst. In fact, the larger the surface area, the higher the contact of the reagents with the catalyst. Moreover, the pore size distribution and the average pore diameter can affect the access and transport of reacting components to the active catalytic surface.

### 2.2. Catalytic Activity of Geopolymers for Transesterification

The number of observable phases and their color are good qualitative indicators of the extension of a transesterification reaction to produce biodiesel. In a typical homogeneous alkali-catalyzed reaction, the mixture starts with two immiscible phases (upper: alcohol; lower: triglyceride feedstock) and ends again with two immiscible phases (upper: biodiesel; lower: crude glycerol). The volume proportion for the maximum biodiesel yield depends on the amount of excess alcohol that is fed into the reactor. For 150% excess, the volume proportion is ~80% for the upper and ~20% for the lower phase. The catalyst cations remain dissolved firstly in the alcohol phase and are then divided between the two product phases. The color of the biodiesel phase is related to the color of the feedstock and usually varies from light yellow to dark gold. On the other hand, the color of crude glycerol is usually darker (reddish to brown). If present, a third top low-viscosity phase in the final mixture is associated with very low conversion grades and with the presence of unreacted alcohol. Another peculiarity of the transesterification process is that glycerol only appears as a product of the reversible reaction that converts monoglycerides into biodiesel, as shown in the set of simplified transesterification reactions ((Equations (1)–(4)):

Triglyceride + Alcohol ⇔ Biodiesel + Diglyceride(1)

Diglyceride + Alcohol ⇔ Biodiesel + Monoglyceride(2)

Monoglyceride + Alcohol ⇔ Biodiesel + Glycerol(3)

1 Triglyceride + 3 Alcohol ⇔ 3 Biodiesel + 1 Glycerol(4)

As observed in the overall reaction (Equation (4)), glycerol is formed in the molar proportion 1:3 to biodiesel. Therefore, the visual presence of a glycerol phase in the post-reaction mixture is a clear indication of its substantial biodiesel content.

**Figure 4:**
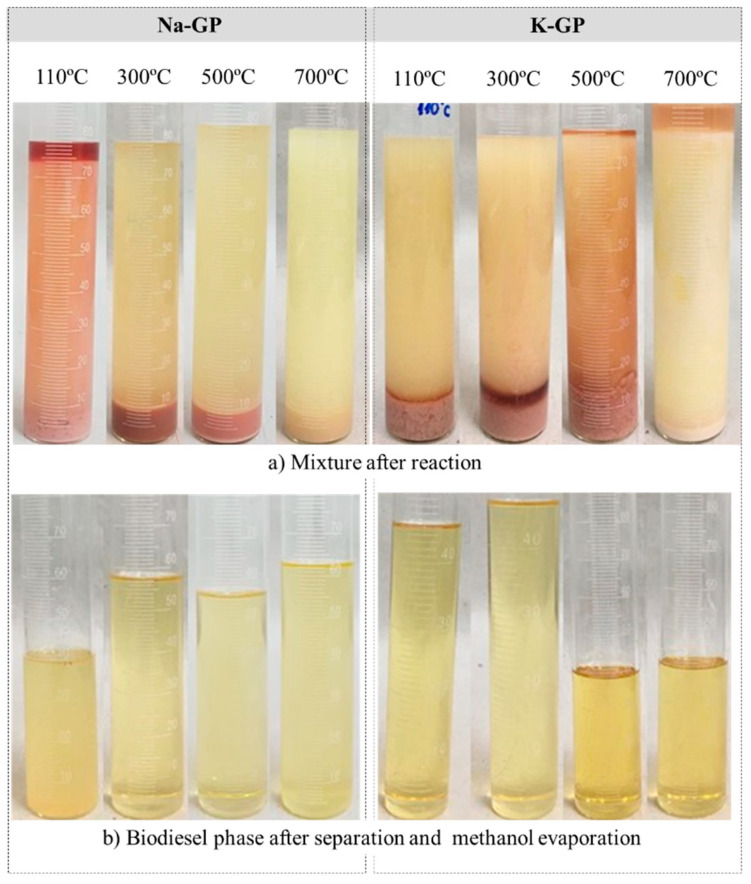
Details of the phases resulting from the transesterification of soybean biodiesel with sodium and potassium geopolymer powders treated at different temperatures. (**a**) Mixture after reaction; (**b**) Biodiesel phase after separation and methanol evaporation.

For heterogeneous-catalyzed systems, such as the one studied in this work, the main qualitative difference is that the catalyst powder remains at the bottom of the reactor due to its higher density, and it is normally mixed or settled within the glycerol phase (see Figure 4a).

Figure 4 shows the details of the post-reaction mixtures and the biodiesel phase after the evaporation of the excess methanol. All the phase and color features that have been previously discussed are present. We can observe in Figure 4a that samples Na-GP, treated at 100 °C, and K-GP, treated at 700 °C, both presented a clear third top phase typical of non-reacted methanol and a low biodiesel yield. On the other hand, Na-GP treated at 700 °C presented only a single light liquid phase, with the catalyst settled at the bottom. The best visual results were those of the Na-GP samples treated at 300 and 500 °C and K-GP treated at 110 and 300 °C, all with a clear reddish bottom glycerol phase. Figure 4b shows samples of the biodiesel phase after evaporation. The light yellow color is typical of biodiesel which has been processed with soybean oil.

**Figure 5:**
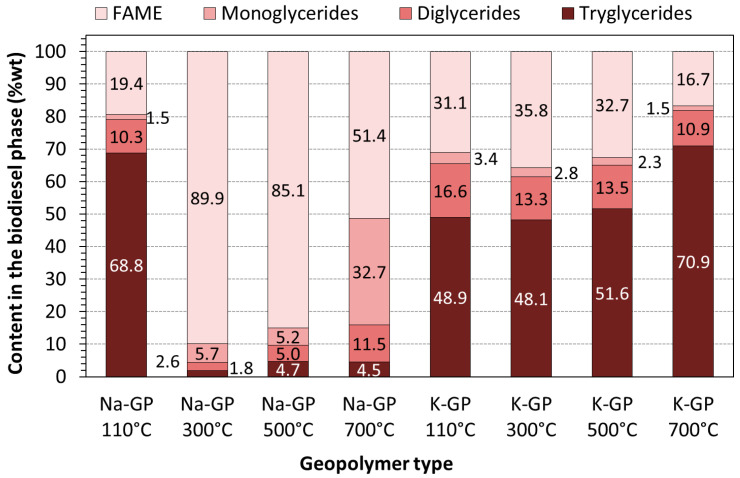
FAME and glycerides contents in biodiesel phase obtained with sodium and potassium geopolymer powders treated at different temperatures.

The catalytic activity of geopolymers Na-GP and K-GP treated at different temperatures was confirmed through the compositional analyses of the biodiesel phase recovered from the transesterification of soybean oil (Figure 4b). The results are presented in Figure 5.

The results confirmed the visual features of the post-reaction mixtures shown in Figure 4. On average, the sodium-based geopolymer powders produced a clearly higher FAME (Fatty Acid Methyl Ester) content than the potassium-based samples. Except for the sample treated at 110 °C, Na-GP provided a relatively high FAME content that reduced from 89.9% to 51.4% as the treatment temperature increased from 300 to 700 °C. It is worth noting that, even for the Na-GP treated at 700 °C, there was a great amount of monoglycerides (32.7%) and very low amount of triglycerides (4.5%), the initial raw material needed for transesterification. This confirms the progress of the reactions ((Equations (1)–(4)) and indicates that a higher FAME content would be achieved for longer reaction times with all Na geopolymers. On the other hand, the K-based geopolymers did not present a clear trend with the treatment temperature but produced consistent levels of FAME content. The chromatogram profiles of Mono, Di and Triglycerides, and the chromatogram profile of biodiesel (FAMEs) are shown in the Supplementary Materials.

No clear correlation was observed between the specific surface area (Table 1) and the composition of the biodiesel phase (Figure 5). The K-GP samples had higher SSA values (28.64–62.54 m^2^/g) but presented lower levels of FAME content (35.8% to 17.6%). Greater amounts of triglycerides were still present in the mixtures (48.9%–70.9%), indicating a modest catalytic activity for the K-GP samples. On the other hand, the sodium geopolymer treated at 110 °C and with the highest SSA value (32.62 m^2^/g) resulted in the lowest FAME content. This poor performance could be due to the presence of residual moisture in this particular sample, and this should be further investigated.

The literature is not conclusive about the performance differences when using either sodium- or potassium-based catalysts. Usually, this depends on the system (triglyceride feedstock, type of alcohol, temperature, etc.) [31,32]. For a typical homogeneous transesterification process with methanol and 1 wt.% catalyst, the same weight amounts of NaOH or KOH in fact imply a higher molar proportion of sodium (and hydroxides) delivered in the reaction due to the differences in the molar masses of Na and K (K/Na = 1.4). Therefore, a 1.4 times higher mass of KOH is theoretically required to match the catalytic effect (time and yield) of NaOH. In the reaction conditions employed in this work, 3 wt.% of either Na-GP or K-GP were used, respectively, with 13.86% of sodium and 21.40% of potassium. The Na or K content actually present in the reaction mixtures was 0.42% and 0.64%, respectively, with minor increases according to the thermal treatment temperature due to the mass loss of hydrates. These amounts are lower than the minimum typically applied in a homogeneous transesterification (~1 wt.%) and, on a molar basis, there were more catalytic sites available for reaction in K-GP compared to Na-GP (K/Na = 1.54). From this perspective, the K-GP would be expected to perform better than the Na-GP, however, this was not observed in this work. One plausible explanation is related to the more effective mass transfer rate of reactants to the catalytic sites inside the geopolymer matrix, as Na-GP had a higher average pore size (17 nm) than K-GP (9 nm), as shown in Table 1. Other aspects should also be further evaluated, including the differences in the basicity of the catalytic sites and the interference caused by metals from the geopolymers leaching into the reaction mixture [33,34]. Further work will also have to be carried out to quantify and compare the amount of gel formed in the Na- and K-geopolymers, as the catalytic performance might be affected by the degree and modality of the incorporation of the alkaline ions into the geopolymer network. Nevertheless, considering the high FAME content achieved in the biodiesel phase (85.1% and 89.9%) for some of the geopolymer samples (Na-GP 300 °C and Na-GP 500 °C), and the fact that minor amounts of the alkali metal ions were leached from the powders into the reaction mixture, it is then reasonable to assume the heterogeneous nature of the catalysis. These issues are presently under investigation by the authors.

Compared to other heterogeneous catalytic systems, the studied geopolymers presented promising levels of FAME content for soybean oil transesterification (85.1%–89.9%), especially considering the mild conditions applied in the experiments (T = 70–75 °C, catalyst = 3 wt.%, methanol to oil ratio = 7.5:1 and t = 4 h). Sahu et al. [8], Singh Chouhan and Sarma [9] and Thangaraj et al. [10] presented comprehensive reviews of alkaline solid catalysts for the transesterification of a variety of oils and under a broad range of conditions. Only a few mixed or doped oxide systems presented similar or better performances when tested in such mild conditions. Sharma et al. [13] achieved high yields (> 95%) by using shorter times (1 h) and with lower amounts of calcium geopolymer catalysts. Nevertheless, those authors used a methanol to oil ratio of 165:1, equivalent to 5418% of methanol, which is completely impractical for proper industrial use.

## 3. Materials and Methods

Two geopolymer compositions were investigated, containing sodium (Na-GP) or potassium (K-GP) as alkaline sources in their formulations. They were prepared using metakaolin (Argical 1200S, Imerys S.A., Paris, France) as an aluminosilicate source and two activating solutions composed by: 1) for Na-GP: sodium silicate (SS2942, Ingessil S.r.l., Montorio, Italy), sodium hydroxide, NaOH (Sigma–Aldrich, Steinheim, Germany) and distilled water; 2) for K-GP: potassium silicate (205K, Tillmanns, Milan, Italy), potassium hydroxide, KOH (Sigma–Aldrich, Steinheim, Germany) and distilled water. The activating alkaline solutions were prepared at least 24 h in advance and were stored at 4 °C. The compositions of the metakaolin and of the sodium and potassium silicates are summarized in Table 2.

The target molar proportions for the geopolymers were as follows: SiO_2_/Al_2_O_3_ = 4.0, SiO_2_/Na_2_O = 3.1, and H_2_O/Na_2_O = 14.2 for the Na-GP system and SiO_2_/Al_2_O_3_ = 4.0, SiO_2_/K_2_O = 3.1 and H_2_O/K_2_O = 14.2 for the K-GP system.

The two formulations were prepared using a mixer (Qualtech Products Industry, Manchester, UK), with a 50 mm 316 stainless steel, alkaline resistant paddle. The metakaolin powder was added to the activating alkaline solution under mechanical stirring for 10 min and 1000 rpm at room temperature. Then, the slurry was poured in a covered plastic mold and placed in an oven at 75 °C for 2 days to complete the geopolymerization reaction. Afterward, the geopolymer blocks were ground and sieved to a particle size between 45 to 125 μm. Finally, the geopolymer powders were dried at 110 °C overnight, to release the remaining water, and they were then heat treated for 1 h in static air (heating rate = 10 °C/min) at three different temperatures (300, 500 and 700 °C).

Table 3 gives the final oxide composition of geopolymers Na-GP and K-GP dried at 110 °C. We can observe that, despite the same target oxide molar ratios of Na_2_O and K_2_O, the weight content of Na and K was not identical as the molar masses (MM) of these two alkalis are different (MW_Na_ = 23 g/mol and MW_K_ = 39 g/mol).

The phase assemblage of the metakaolin and the heat-treated geopolymers was investigated by X-ray diffraction analysis (XRD) using an X-ray diffractometer (D8 Advance, Bruker Corporation, Karlsruhe, Germany) with Cu-Kα radiation, operated at 40 kV and 40 mA with a 0.05° step width, a scanning range of 10–70° and a scanning speed of 3 s/step. The software Match!, version 1.4d (Crystal Impact, GbR, Bonn, Germany), supported by the PDF-2 Powder Diffraction File by ICDD (International Center for Diffraction Data, Newtown Square, PA, USA), was used to identify the crystalline phases. Thermogravimetry analysis and differential thermal analysis (DTA/TG STA409, Netzsch, Selb, Germany) was carried out in static atmospheric air, from room temperature to 1400 °C, with a heating rate of 10 °C/min. The specific surface area (SSA) of the samples was measured by N_2_ adsorption at liquid nitrogen temperature, using the multi-point Brunauer–Emmett–Teller (BET) method and a Quantachrome Autosorb iQ (Quantachrome Instruments, Boynton Beach, FL, USA). Prior to the analysis, the samples were degassed for approximately 17 h under reduced pressure at 120 °C. After the BET data were obtained, the total pore volume, the average pore diameter and the pore size distribution were calculated using the Barrett–Joyner–Halenda (BJH) method. All samples had a particle diameter < 125 μm and were dried at 110 °C overnight before the analyses.

**Figure 6:**
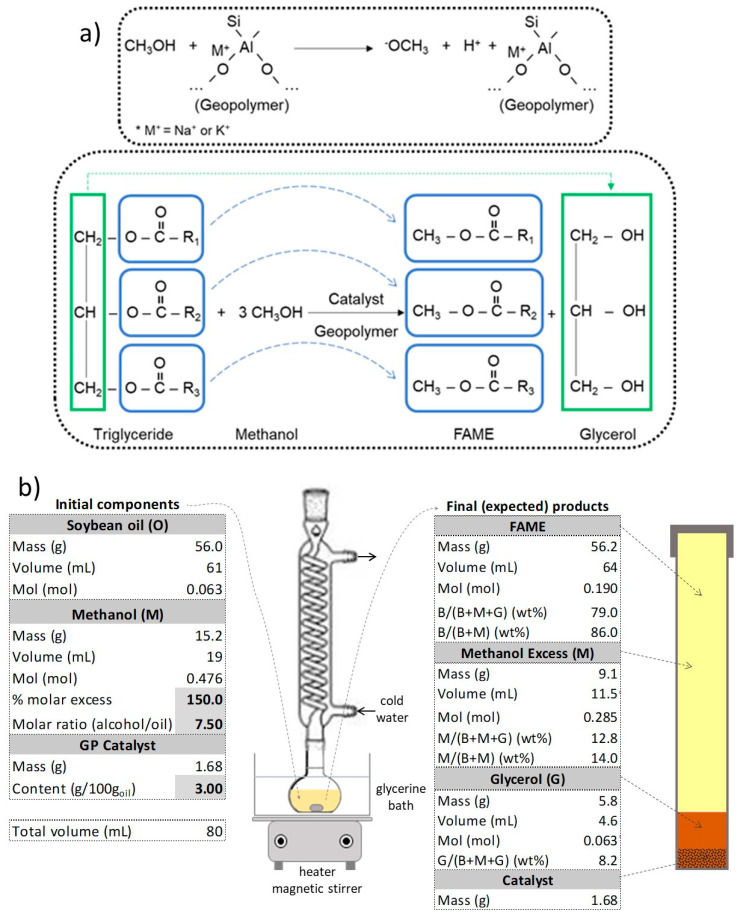
(**a**) Schemes of methoxide formation and transesterification reaction; (**b**) recipe for methyl transesterification of soybean oil and expected products using Na and K geopolymer catalysts.

The transesterification reaction between the oil and alcohol was conducted using commercial refined soybean oil (Soya, Bunge, Mato Grosso, Brazil) and methanol (Applychem PanReac, Darmstadt, Germany). The reaction was carried out under a vigorous agitation in a 100 mL flask, linked to a vertical condenser with cold water in order to force the total reflux of methanol in the flask. The reactor was immersed in a glycerin bath to keep the mixture temperature in the 70–75 °C range. The reaction time was 4 h for all the tests. The proportions adopted for the transesterification were a molar ratio of methanol to oil of 7.5:1 (150% excess methanol) and 3% of catalyst (*w*/*w* oil). After the reaction time, the mixture was cooled and centrifuged to separate the produced phases (biodiesel and glycerol). The separated biodiesel phase was evaporated at 100 °C to eliminate the excess methanol. No washing was performed prior to the analysis, in order to preserve the whole glycerides and FAME profiles in the biodiesel phase. Figure 6a presents the scheme of the expected interaction between methanol and the geopolymer to form methoxide ions and between methanol and the triglycerides to produce fatty acid methyl esters (FAME). Figure 6b summarizes the recipe used for transesterification and the expected products for maximum FAME content.

The official methods proposed by the Brazilian Technical Standards Association (ABNT NBR 15908) [35] and the European Standards (EN 14103) [36] were respectively used to quantify the glycerides (MG, DG and TG) and the methyl ester in the biodiesel phase. Both determinations were made by GC-FID (gas chromatography with flame ionization detector, Model GC2010, Shimadzu, Japan).

For the glyceride quantification, the chromatographic system was configured to separate and identify monoglycerides (MG), diglycerides (DG) and triglycerides (TG) with a Crossbond^TM^ 5% Phenyl/95% dimethylpolysiloxane capillary column (Zebron ZB-5HT, 30 m × 0.32 mm × 0.1 mm—Phenomenex, Torrence, CA, USA), with on-column injection. The sample aliquot (0.5 mL) was injected without split ratio and a helium carrier gas (2.0 mL min^−1^) was used. The initial temperature in the capillary column was 50 °C (1 min), and increased to 180 °C at 15 °C/min, 230 °C at 7 °C/min and finally to 380 °C at 20 °C/min, with a 10 min holding time. The injector and detector temperatures were 380 °C and the sample was prepared using heptane 99%. For the quantification of the fatty acid methyl esters (FAME), the chromatographic system was configured to separate and identify free fatty acids (FFA) with a Crossbond^TM^ polyethylene glycol capillary column (Stabilwax, Restek, Bellefonte, PA, USA). The initial temperature was 60 °C and was increased at 10 °C/min to 200 °C and at 5 °C/min to 240 °C, with a 7 min holding time. The injector and detector temperatures were 350 °C and the samples were prepared using C19:0 (methyl nonadecanoate) with internal standard e toluene 99%. The sample aliquot (1.0 mL) was injected using split flow (100 mL min^−1^) and a helium carrier gas (1.5 mL min^−1^) was used.

The performance of the transesterification reaction in biodiesel production has been reported in a variety of ways in the literature [37]. In this work, the content of FAME in the biodiesel phase was quantified through Equation (5):

(5) $$\mathrm{FAME}\;\mathrm{content}\;\left(\%\right)=\frac{\mathrm{Mass}\;\mathrm{of}\;\mathrm{FAME}\;\mathrm{calculated}\;\mathrm{by}\;\mathrm{GC}\;\mathrm{analysis}}{\mathrm{Mass}\;\mathrm{of}\;\mathrm{biodiesel}\;\mathrm{phase}\;\mathrm{analyzed}}100\%$$

## 4. Conclusions

Sodium and potassium-based geopolymer powders were tested as heterogeneous catalysts for the methyl transesterification of soybean oil in order to produce FAME (biodiesel). Despite the same target molar oxide proportions, the resulting potassium geopolymers possessed higher SSA and lower pore size values, attributable to differences in the viscosity of the activating solutions and in the metakaolin conversion before setting. The increase in the heat treatment temperature (from 110 to 700 °C) caused a reduction in the SSA for both formulations, but this was more severe for the sodium formulation due to its higher thermal shrinkage. The Na-based geopolymers performed better for the methyl transesterification of soybean oil, and the reason was ascribed to the higher average pore diameter that enhanced access to the catalytic sites and to the higher amount of catalytic sites in the Na-based formulation compared to the K-based one, on a molar basis. No clear relationship between SSA values and the biodiesel yield could be ascertained. High levels of FAME content of 85.1% and 89.9% were achieved with the sodium geopolymers that were treated at 500 and 300 °C, respectively. These values are quite promising considering the very mild reaction conditions used in the experiments compared to when using other heterogeneous catalysts which have been reported in the literature.

## Figures and Tables

**Table 1:** Physical features of the geopolymer powder samples.

Sample	Treatment Temperature (°C)	Specific Surface Area (m²/g)	Total Pore Volume (cm^3^/g)	Average Pore Diameter (nm)
Na-GP	110	32.62	0.30	17.2
	300	31.41	0.31	17.2
	500	27.43	0.28	17.2
	700	6.34	0.08	17.2
K-GP	110	62.54	0.33	9.5
	300	42.18	0.29	9.4
	500	29.85	0.34	9.5
	700	28.64	0.31	9.4

**Table 2:** Composition of commercial raw materials used to prepare the geopolymers.

Raw Material	SiO_2_ (wt.%)	Al_2_O_3_ (wt.%)	Na_2_O (wt.%)	K_2_O (wt.%)	H_2_O (wt.%)	Other (wt.%)
Metakaolin (Argical 1200S)	55.00	39.00	<1.00	<1.00	-	5
Na silicate (Ingessil 2942)	28.35	-	9.77	-	61.88	-
K silicate (T205K)	57.26	-	-	27.24	15.5	-

**Table 3:** Oxide composition on a dry basis of the sodium-based (Na-GP) and potassium-based (K-GP) geopolymers tested in this work.

Oxide Component	Na-GP (wt.%)	K-GP (wt.%)
SiO_2_	54.83	50.05
Al_2_O_3_	23.26	21.23
Fe_2_O_3_	1.07	0.98
TiO_2_	0.89	0.82
Na_2_O	18.68	0.27
K_2_O	0.30	25.77
CaO + MgO	0.36	0.33
LOI	0.60	0.54
Total	100.00	100.00
Na (%wt.)	13.86	0.20
K (%wt.)	0.25	21.40

## Supplementary Materials

The following are available online: Figure S1: Chromatogram profiles of Mono (1 and 2); Di (3 and 4); Triglycerides (5) obtained through ABNT NBR 15908 in biodiesel yields with sodium geopolymer powders treated at different temperatures; Figure S2: Chromatogram profiles of Mono (1 and 2); Di (3 and 4); Triglycerides (5) obtained through ABNT NBR 15908 in biodiesel yields with potassium geopolymer powders treated at different temperatures; Figure S3: Chromatogram profile of biodiesel (FAMEs) obtained through EN 14103 using with sodium geopolymer powders treated at different temperatures; Figure S4: Chromatogram profile of biodiesel (FAMEs) obtained through EN 14103 using with potassium geopolymer powders treated at different temperatures.
