# Supplementary material for: Biodiesel Processing Using Sodium and Potassium Geopolymer Powders as Heterogeneous Catalysts
Source: Molecules. 2020 Jun 19;25(12):2839. doi: 10.3390/molecules25122839 (PMC7356640; doi:10.3390/molecules25122839)

Supplementary Material

Biodiesel Processing Using Sodium and Potassium Geopolymer Powders as Heterogeneous Catalysts

Renata F. Botti [1,*], Murilo D.M. Innocentini [2], Thais A. Faleiros [2], Murilo F. Mello [2], Danilo L. Flumignan [3,4], Leticia K. Santos [3], Giorgia Franchin [1] and Paolo Colombo [1,5]

[1] Department of Industrial Engineering, University of Padova, via Marzolo 9, 35131 Padova, Italy; giorgia.franchin@unipd.it (G.F.); paolo.colombo@unipd.it (P.C.)

[2] Course of Chemical Engineering, University of Ribeirão Preto (UNAERP), 14096-900 Ribeirão Preto, São Paulo, Brazil; minnocentini@unaerp.br (M.D.M.I.); thaisfaleiros1999@hotmail.com (T.A.F.); murilofmello@hotmail.com (M.F.M.)

[3] Institute of Chemistry, Center for Monitoring and Research of the Quality of Fuels, Biofuels, Crude Oil and Derivatives–CEMPEQC, São Paulo State University (UNESP), 14800-900 Araraquara, São Paulo, Brazil; flumignan@ifsp.edu.br (D.L.F.); leticiaksantos@yahoo.com (L.K.S.)

[4] Mato Grosso Federal Institute of Education, Science and Technology-Campus Cuiabá, Rua Profa. Zulmira Canavarros, 95, Centro, 78005-200 Cuiabá, Mato Grosso, Brazil

[5] Department of Materials Science and Engineering, The Pennsylvania State University, University Park, PA 16802, USA

* Correspondence: renata.botti@unipd.it; Tel.: +39 049 8275513

Figure S1. Chromatogram profiles of Mono (1 and 2); Di (3 and 4); Triglycerides (5) obtained through ABNT NBR 15908 in biodiesel yields with sodium geopolymer powders treated at different temperatures.

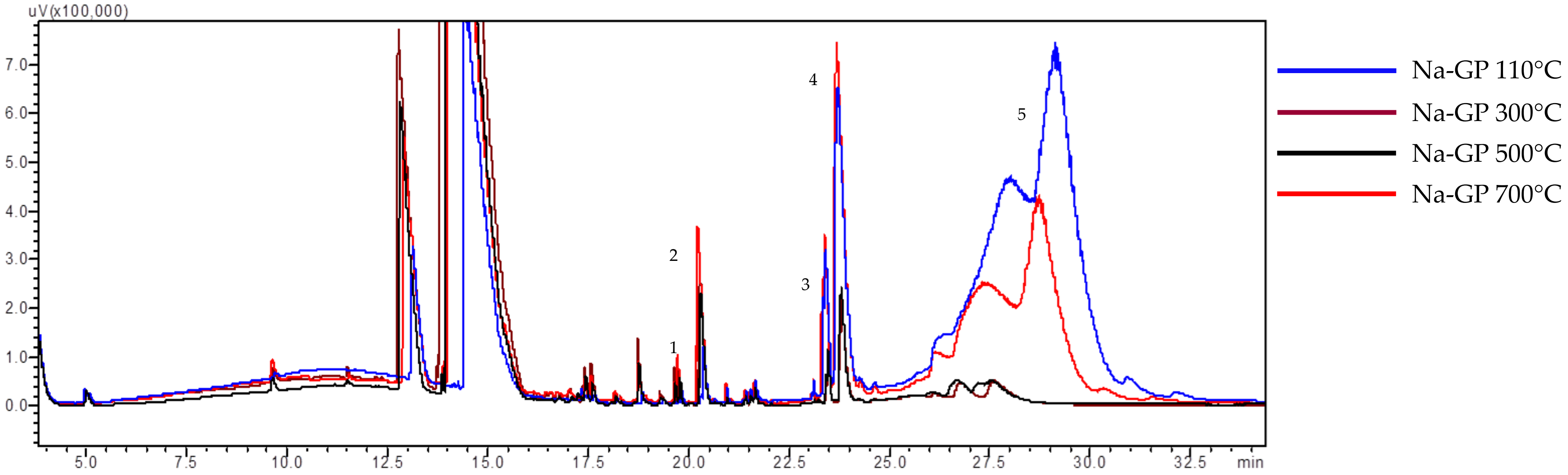

Figure S2. Chromatogram profiles of Mono (1 and 2); Di (3 and 4); Triglycerides (5) obtained through ABNT NBR 15908 in biodiesel yields with potassium geopolymer powders treated at different temperatures.

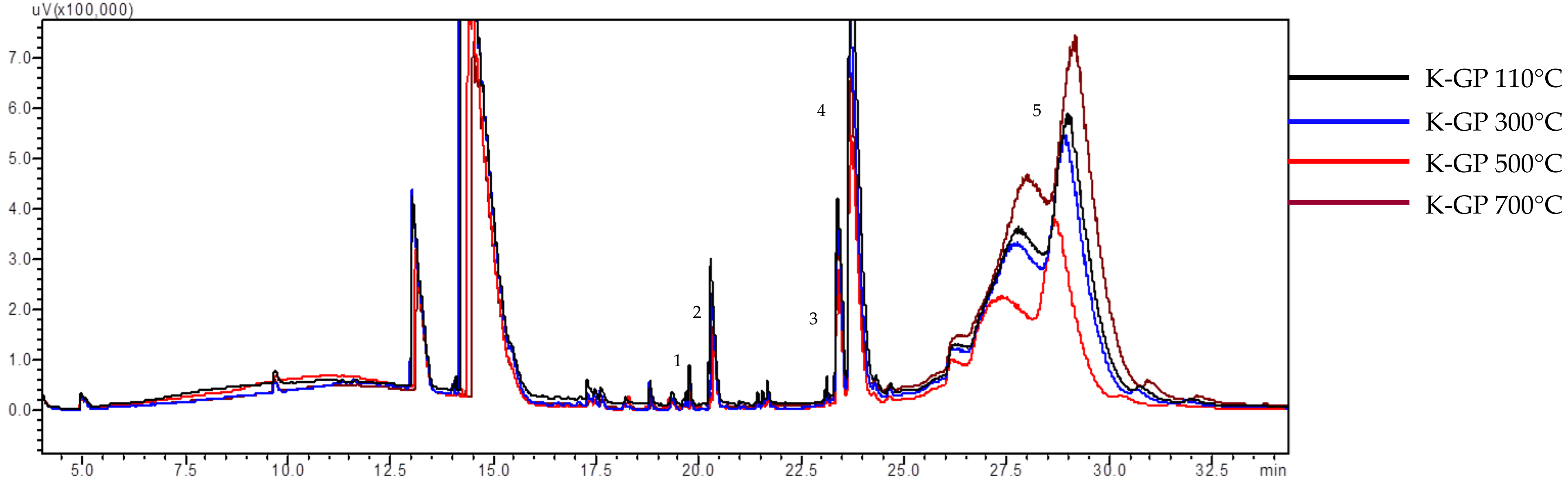

Figure S3. Chromatogram profile of biodiesel (FAMEs) obtained through EN 14103 using with sodium geopolymer powders treated at different temperatures.

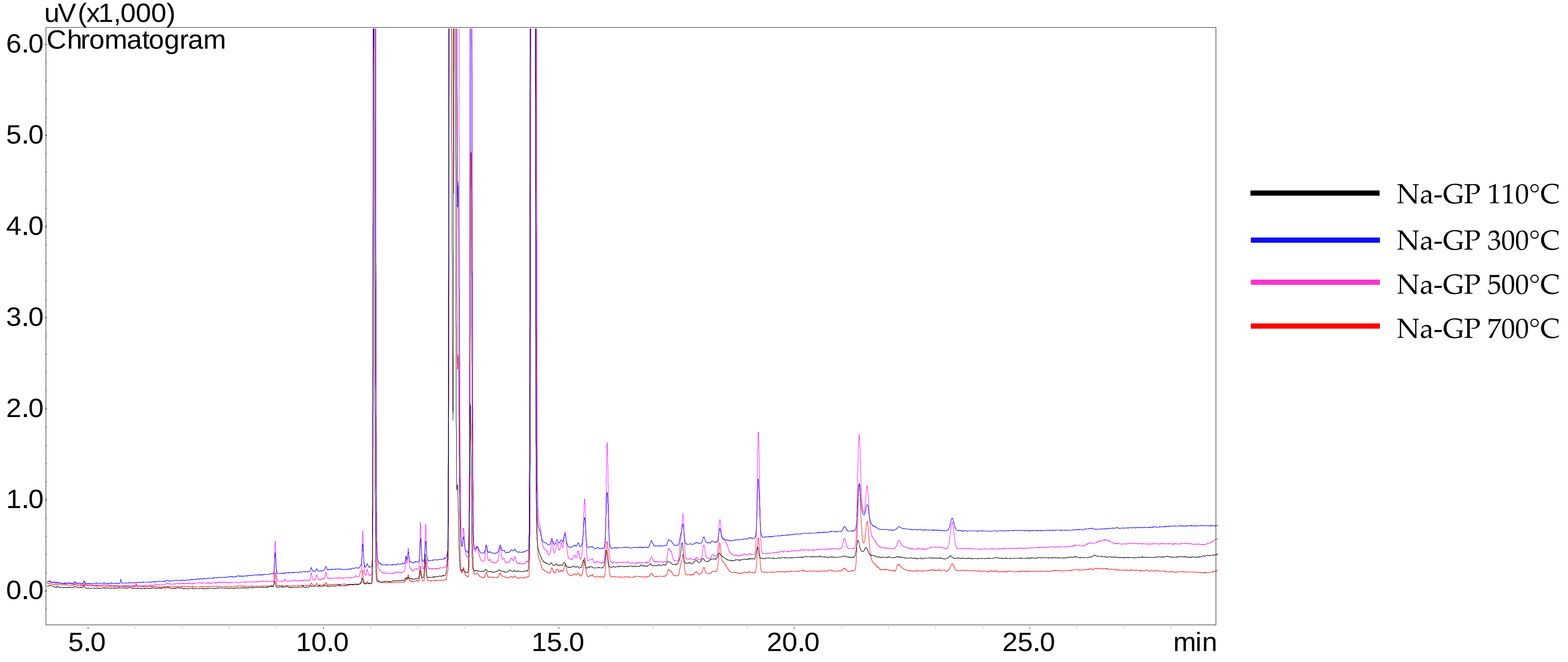

Figure S4. Chromatogram profile of biodiesel (FAMEs) obtained through EN 14103 using with potassium geopolymer powders treated at different temperatures.

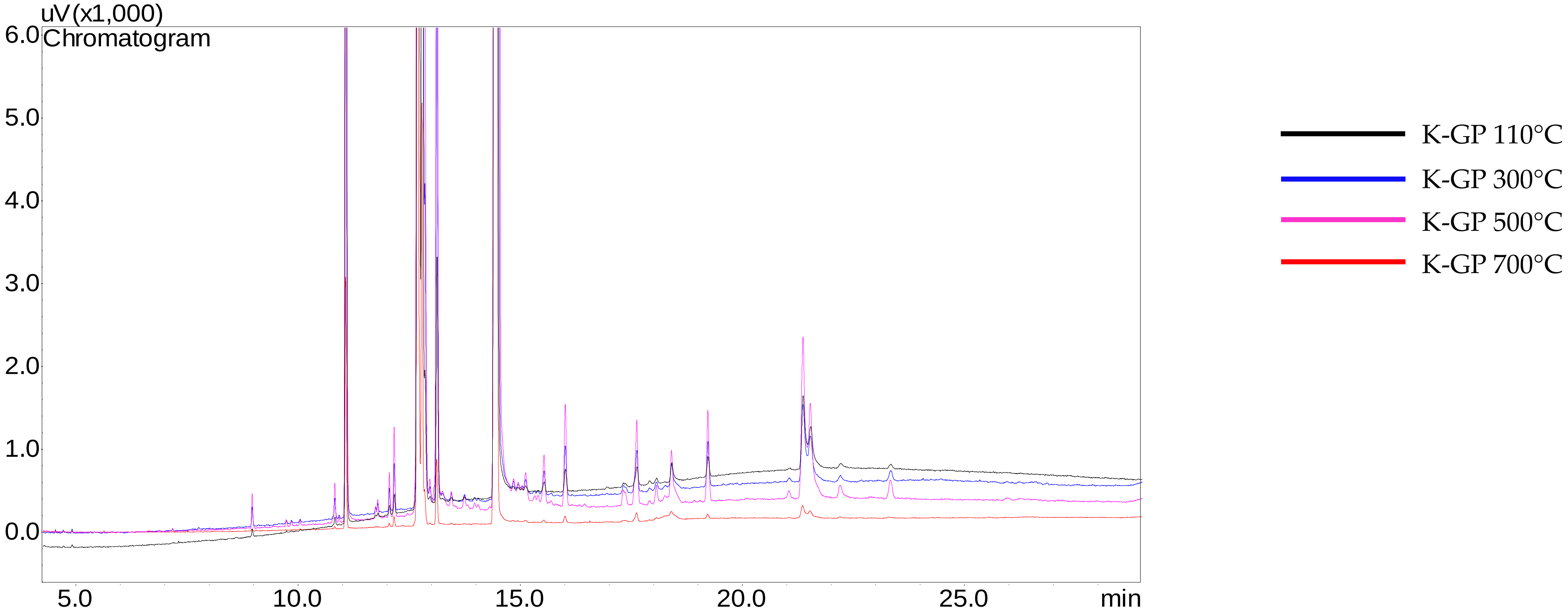

Supplement: Supplementary file 1 [file molecules-25-02839-s001.pdf]
